# A Case of Puerperal Eclampsia

**Published:** 1880-12

**Authors:** J. W. Keene


### A CASE OF PUERPERAL ECLAMPSIA.

REPORTED BY J. W. KEENE, M. D.

On Sept. 9th, 1880, I was summoned in haste to see Mrs. D., who was said to be in a fit. On arrival the patient was found to be a young compactly built brunette of 22 years, recently married, and now about six months pregnant with her first child. The limbs were highly œdematus, the dorsa of the feet smooth and shining. It was said that the swelling had been worse, but had been treated by a physician in the town from which the patient had just removed, who succeeded in reducing it somewhat. I could not learn that the urine was examined at that time.

The first convulsion occurred at 10 P. M.; another occurred at 11 P. M., before I reached the house; and they continued to occur at intervals of about an hour until 10 A. M. the next day. During these twelve hours she had ten convulsions. The bromides and chloral were given in large doses, but their effect was unsatisfactory. Chloroform was administered, beginning at about the time a convulsion was due and pressing it to the full surgical extent, but without arresting the attacks. No attempt at venesection was made, as the measure had proved abortive in previous experience from inability to establish a flow of blood.

The os was found sufficiently dilated to admit the finger, but there were no uterine contractions. The fontanelle could be reached, and it was observed that there was no perceptible pulsation of the brain.

Dr. B. H. Daggett was called in consultation, and after deliberation it was decided not to attempt artificial delivery at present, but to await events as the death of the fœtus was not assured.

Efforts to check the convulsions proving futile by the means above enumerated, the patient at 10½ A. M. was chloroformed thoroughly and kept in that state for three and one-half hours or until 2 P. M. During this time chloral hydrate was administered hypodermically 1 gram every 45 minutes, alternating with 1.5 grams of bromide of potassium administered per rectum. In all 5 grams of chloral hydrate and 9 grams of bromide were administered. At 2 P. M. the anesthetic was withdrawn. The patient lay for several hours in a profound comatose state. There were no more convulsions. She at length regained consciousness and conversed with her friends during the evening.

During this period no symptoms of chloral poisoning were manifest except the coma, which of course was not observed until the removal of the anesthetic. The pulse and respiration showed no signs of flagging. Bromide and chloral were given for some time moderately, and the patient kept on the verge of coma until the next day, when the chloral was discontinued, and later the bromide also. The suppression of urine was complete until the third day. Albumen in large amount was contained in the first scanty passage. No motion of the fœtus was felt afterward, and it was delivered on the tenth day by Dr. Dorland, who was summoned during my detention elsewhere. There was nothing remarkable about the fœtus or its delivery. It was but slightly macerated. The patient made a good recovery. The albumen gradually disappeared from the urine under the usual remedies. Two troublesome abscesses formed at points of puncture of the hypodermic needle, one of which did not entirely heal until the expiration of two months. No albumen was seen after six weeks.

